# A comparison of symptoms and quality of life before and after nasal septoplasty and radiofrequency therapy of the inferior turbinate

**DOI:** 10.1186/s12901-017-0050-z

**Published:** 2018-01-26

**Authors:** Ann Helen Nilsen, Anne-Sofie Helvik, Wenche Moe Thorstensen, Vegard Bugten

**Affiliations:** 1 Department of Ear, Nose and Throat, Head and Neck Surgery, St Olavs University Hospital, 7006 Trondheim, Norway; 2 Institute of Public Health and General Practice, Norwegian University of Science and Technology, 7006 Trondheim, Norway; 3 Department of Neuromedicine and Movement Science, Norwegian University of Science and Technology, 7006 Trondheim, Norway

**Keywords:** Nasal obstruction, Surgery, Health related quality of life, Septum deviation, Inferior turbinate hypertrophy

## Abstract

**Background:**

The primary goal of this study is to compare pre- and postoperative symptoms and health related quality of life (HQOL) in 57 patients who underwent septoplasty (group-1), 56 patients who underwent septoplasty combined with radiofrequency therapy of inferior turbinates (RFIT) (group-2) and 58 patients who underwent RFIT alone (group-3). The secondary goal is to investigate if the change in symptoms and HQOL differed between these three patient groups after surgery.

**Methods:**

All patients reported symptoms on a visual analogue scale (VAS) and HQOL on Sino-Nasal-Outcome-Test-20 (SNOT-20) and Short-Form-Health-Survey-36 (SF-36) before and 6 months after surgery. The pre- and postoperative scores and improvement were compared within and between the three patient groups.

**Results:**

Preoperatively the three patient groups had a fairly similar symptom burden and HQOL, except for group-1 which reported more symptoms of oral breathing than group-3 *(p < 0.01)* and group-3 which reported more problems in the ear/facial--subset of SNOT-20 and in the general-mental-health-domain of SF-36 than group-1 *(p < 0.01)*.

Postoperatively all patient groups reported improved symptom scores of nasal obstruction, nasal discharge, snoring, oral breathing and reduced general health *(p < 0.01),* and better HQOL *(p < 0.05).* Patients in group-2 had less symptoms of nasal obstruction than group-3 *(p < 0.05)*. Postoperative symptom score for nasal obstruction was 29.1 (SD67.6) in group-1, 27.5 (SD22.5) in group-2 and 37.2 (SD24.8) in group-3. Revision cases reported more nasal obstruction postoperatively; 41.3 (SD27) than non revision cases; 28.6 (SD24) *(p < 0.01).*

The HQOL after surgery was about the same in all three patient groups, but we found that patients with comorbidities as sleep apnea and asthma reported worse HQOL than other patients *(p < 0.01).*

**Conclusion:**

Surgical treatment of nasal obstruction led to less symptoms and better HQOL for all three patient groups. Comparing the postoperative scores between the patient groups we find that all groups reached the same level of HQOL. Regarding symptoms, the patients who underwent septoplasty combined with RFIT reported postoperatively less nasal obstruction than patients who underwent RFIT alone which may indicate that a combined procedure of septoplasty and RFIT is better than RFIT alone to treat nasal obstruction. Furthermore, revision cases, patients with sleep apnea and asthma patients seem to have poorer outcome after surgery than other patients. Both disease specific and general QOL instruments add valuable information for identifying factors influencing outcome.

**Electronic supplementary material:**

The online version of this article (10.1186/s12901-017-0050-z) contains supplementary material, which is available to authorized users.

## Background

Patients with symptoms of nasal obstruction frequently consult an otorhinolaryngologist [1]. Nasal obstruction negatively affects patients’ quality of life (QOL) [1–3]. Sustained nasal obstruction may have anatomical or structural causes such as deviation of the nasal septum or inferior turbinate hypertrophy (ITH) [4], but chronic diseases such as chronic rhinosinusitis (CRS) and allergic rhinitis (AR) [5, 6] also cause nasal congestion and reduced nasal airflow.

Nasal septal deviation has a prevalence ranging from 19% to 65% due to different definition criteria [7, 8]. Characteristic symptoms of a deviated septum can be nasal obstruction, nasal discharge, sneezing, snoring, oral breathing, and sleep apnea [9]. Some patients with a deviated septum have troublesome symptoms that lead to surgery.

ITH can cause nasal airway obstruction and affects 10–20% of Europe’s adult population [10]. ITH can occur in isolation or in combination with deviation of the septum. Normally, patients are treated medically with anti-histamines, topical decongestants and corticosteroids; surgery is reserved for refractory cases [11, 12]. During the last decade, radiofrequency therapy of the ITH (RFIT) has been performed more frequently in combination with septoplasty [1] or as a single approach to reduce nasal obstruction in patients with ITH [13–15]. Various surgery techniques have been used to reduce ITH, but radiofrequency coblation and microdebrider-assisted turbinoplasty are common methods because they are easy to perform [12, 16]. In the literature, there is no clear consensus on the optimal surgical method, optimal selection of patients or expected improvement in symptoms [16–20].

Even if objective measures regularly are being used assessing nasal patency [21], QOL measures are an important guide for measuring the efficacy of surgical interventions, and have thus been used with increasing frequency in recent years within several sino-nasal disorders [11, 22, 23]. There are a large numbers of definitions of QOL. Health related quality of life (HQOL) is the most frequently used approach in epidemiological and clinical health research [24]. HQOL captures aspects of an individual’s subjective experience of QOL related to health, disease, disability and impairment and the effects of medical treatment [25]. HQOL is subjective and a multidimensional construct [24] and highlight also the social and psychological consequences of diseases, as the health-care interventions aim to improve [26].

Contradictory results have been reported from studies depending on whether they studied improvement in symptoms or HQOL in patients undergoing surgery for chronic nasal obstruction [9, 14, 27, 28]. To our knowledge, we have not found studies comparing these three differernt diagnosistic groups, and few studies have used both Sino-Nasal OutcomeTest-20 (SNOT-20) and the Short- Form Health Survey 36 (SF-36) to explore whether sino-nasal aspects and more general aspects of HQOL have improved in patients undergoing septoplasty and RFIT [27]. In daily practice it is a challenge tailoring the right patients for the optimal surgery. Patients with clinical significant septal deviation, clinical septal deviation combined with ITH or ITH without significant clinical septum deviation present with the same cardinal symptom; nasal obstruction. There is little evidensbased knowledge guiding the surgeon in decision making. Selection of optimal surgery is based on each surgeons clinical assessment. Assessing the symptom- and HQOL score of the patients may help the surgeons to decide optimal treatment.

The primary aim of this prospective registry-based outcome study was to compare symptoms and HQOL before and after surgery in three patient groups; those who underwent septoplasty alone, septoplasty combined with RFIT and RFIT alone. The secondary aim was to investigate if the change in symptoms and HQOL differed between these three patient groups after surgery.

## Material and methods

### Ethics, consent and permissions

This prospective registry study was conducted during the period from January 2012 to April 2015 and was approved by the Committee for Medical Research Ethics in Norway, 2015–367/REK NORD. All patients signed a written consent prior to inclusion in the study.

### Materials

All patients were referred from general practitioners, private otorhinolaryngologists, or local hospitals in the region to assessment for surgical treatment at the ENT department at St Olavs University Hospital. All patients were examined at the outpatient clinic by a varity of surgeons.

Diagnosis was based on anterior rhinoscopy and nasal endoscopy combined with patients` symptoms. Nasal decongestants was not used in the diagnostic. The diagnoses were based on the International Classification of Diseases (ICD-10) codes J34.2 (septum deviation) and J34.3 (ITH). When there was indication for septoplasty alone, septoplasty in combination with RFIT or only RFIT the patients were asked to participate in the study.

Inclusion criteria were a deviated septum, a deviated septum in combination with ITH or ITH alone without clinical significant septum deviation with presenting symptoms of chronic nasal obstruction, symptoms lasting at least three months and persistent symptoms after medical management.

Exclusion criteria were age less than 18 years, difficulty in interpreting the questionnaires due to language or cognitive problems, pregnancy, ongoing cancer treatment, granulomatosis with polyangiitis, cystic fibrosis, Kartagener syndrome, sarcoidosis or ciliar dyskinesia.

**Fig. 1:**
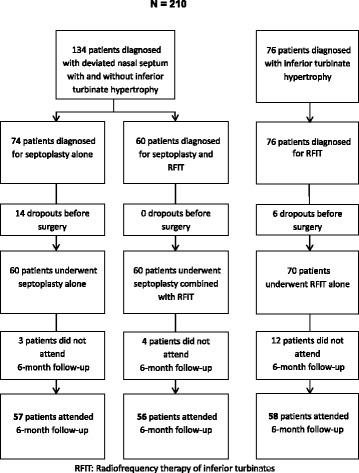
Flow chart

We included 210 patients. Due to dropouts before surgery (20 patients) and loss of follow-up (17 patients), the total sample of this study was 171 patients, where 57 patients underwent traditional cartilage-preserving septoplasty alone, 56 patients underwent a combination of septoplasty and RFIT, and 58 patients underwent RFIT alone (Fig. 1).

### Methods

#### Symptoms and HQOL

The patients’ symptoms were indicated on 100 mm visual analog scales (VAS) where 0 mm represents no symptoms and 100 mm represents symptoms “as troublesome as possible”. Symptoms reported were nasal obstruction, nasal discharge, sneezing, snoring, oral breathing and reduced general health [29]. The symptom severity is considered mild between 0 and 30, moderate from 30 to 70 and severe from 70 to 100 [30].

The Sino-Nasal Outcome Test-20 (SNOT-20) questionnaire was used to assess HQOL more specifically related to the sino-nasal outcome. It has been validated in patients with chronic rhinosinusitis [31, 32], and used to assess sino-nasal outcome in relation to other diseases such as asthma [33], cystic fibrosis [34], skull base tumors [35] and in healthy individuals [33].

The patients graded 20 items on a scale from 0 (no problem) to 5 (problem as severe can be). The total SNOT sum score for each patient was defined as the mean value of the response to the 20 items. The questionnaire is divided into four subsets [23]. The first subset is related to the nose issues, the second subset to ear and face issues, the third subset to sleep quality and the fourth subset to psychological issues. Questions about cough and waking up tired are separate entities and do not belong to any subset. A mean score was calculated for each of the subsets.

More general aspects of HQOL were assessed with the Norwegian validated version of the Short-Form-Health-Survey 36 (SF-36) [36–38]. SF-36 can be used to compare HQOL profiles for groups differing in diagnosis, disease severity or treatment regimen and monitor transitions in health status over time for diverse groups [37]. It containes 36 questions belonging to eight domains of HQOL; physical function, restriction in physical role, restriction in emotional role, vitality, social function, bodily pain, mental general health. The eight domain respective scales are gathered in two summary scales, divided into physical and mental health.

### Statistics

We used PASW Statistics, version 23 for Windows (SPSS Inc. Chicago, Illinois) for statistical analysis. The mean value ± SD was used to describe symptoms and HQOL. Categorical and ordinal variables were analyzed with the Pearson chi-square test or Fisher exact test depending on sample size. All data regarding symptoms and HQOL at baseline and follow-up were not normally distributed. For comparative analyses of continuous variables we used the Mann-Whitney and Wilcoxon signed ranked test. *P*-values less than 0.05 were considered statistically significant. Power calculations showed that with 40 patients in each group and a significance level of 0.05 (alpha), we were able to detect a difference in SNOT-20 of 0.6 (SD1.2) between the groups with 80% power. With 100 participants in each group and the same assumptions as above, we would be able to detect a difference in SNOT-20 of 0.4.

## Results

The baseline characteristics of the patients who underwent septoplasty (group 1), septoplasty with RFIT (group 2) or RFIT alone (group 3) did not differ in demographic or medical characteristics except for more men in group 1 than in group 3 *(p < 0.01)* (Table 1).

**Table 1:** Demographic and medical characteristics at baseline

	Total *N* = 171	Group 1 *N* = 57	Group 2 *N* = 56	Group 3 *N* = 58
Mean age, years	38,6 (13,7)	36,5 (14,0)	40,5 (14,4)	38,9 (12,7)
Mean BMI, kg/m^2^	27,2 (4,63)	26,3 (4,47)	27,9 (4,68)	27,5 (4,70)
Sex (m/f)	127/44	48/9	43/13	36/22
Smoke daily	18	3	7	8
Allergy	71	26	22	23
Asthma	25	7	9	9
Sleep apnea	38	9	16	13
Previous surgery	38	12	11	15

Abbreviations: Group 1, septoplasty; Group 2, septoplasty combined with radiofrequency therapy of inferior turbinate (RFIT); Group 3, RFIT only; BMI, body mass index. Revision cases: patients having prior surgery of septoplasty, septoplasty combined with RFIT or RFIT alone

### Surgical procedures and postoperative care

*Patient group 1:* The mean duration of surgery in the 57 patients who underwent traditional cartilage-preserving septoplasty alone was 71 min (SD 28). Of these patients, 11 patients had local anesthesia, 46 patients had a silastic plate bilaterally for support and to prevent adhesions postoperatively and 36 patients had a nasal packing to prevent bleeding and hematoma of the septum for 2 days.

*Patient group 2:* The mean duration of surgery in the 56 patients who had a combination of septoplasty and RFIT was 73 min (SD 32). Of these patients, 44 patients had a silastic plate bilaterally postoperatively and 44 patients had a nasal packing for 2 days. Septoplasty combined with RFIT was performed under general anesthesia with the CelonProBreath® bipolar coagulation electrode (Celon AG medical instruments 2003 Rheinstrasse 8, D-14513 Teltow/Berlin, Germany). The power setting was 15 watts and exposure time ranged from 5 to 15 s with varying applications in each turbinate.

*Patient group 3:* For the 58 patients who underwent RFIT alone, the mean duration of surgery was 13 min (SD 7) and 57/58 had surgery under local anesthesia. RFIT was done with the Sutter system BM-780 II (Sutter medizintechnik GMBH Tullastrasse 87, 79,108 Freiburg, Germany) AutoRF setting, power adjustment 2; exposure time ranged from 5 to 9 s in each application. The number of applications in each turbinate was assessed by the surgeon.

No treatment allocation, randomization or other attempt to modify treatment was made. The procedures were performed by 14 different surgeons: six consultants and eight senior registrars at St Olavs Hospital. The nasal packing was removed by a nurse in the outpatient clinic or by the patients themselves; the plates were taken out by the surgeon 1 week after surgery. The 6 months follow up was done at the outpatient clinic. The patients filled out the questionnaires alone and handed them to a trained nurse.

### Symptoms on VAS before and after surgery

Preoperatively the symptom scores on VAS were fairly similar. Group 3 reported less symptoms of oral breathing than patients in group 1 (*p* < 0.01) (Table 3). Nasal obstruction was the most bothersome preoperative symptom in all three groups.

Six months after surgery all patient groups had significant improvement of all symptoms, except for sneezing in group 3. Patients in group 1 and 2 had significantly greater improvement in symptoms than patients in group 3 (*p* < 0.04, *p* < 0.01), especially for the symptom of nasal obstruction (*p* < 0.04) (Table 3). The improvement in nasal obstruction was 40.5 (SD34) mm for group 1, 44.6 (SD26) mm for group 2 and 29.5 (SD32) mm for group 3.

**Fig. 2:**
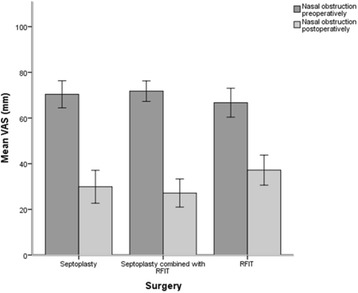
Nasal obstruction preoperatively and 6 months postoperatively. Values presented as mean, 95% CI

Postoperatively patients in group 3 reported significantly more symptoms of snoring (*p* < 0.03) than group 1. Group 3 reported more symptoms of nasal obstruction (*p* < 0.04) and sneezing (*p* < 0.02) than group 2. The symptom score for nasal obstruction was 29.1 (SD27.0) mm in group 1, 27.5 (SD22.5) mm in group 2 and 37.2 (SD24.8)mm in group 3 (Table 2 and Fig. 2).

**Table 2:** Symptoms and HQOL preoperatively and 6 months postoperatively

	Group 1	Group 1		Group 2	Group 2		Group 3	Group 3	
	Pre	Post		Pre	Post		Pre	Post	
	N = 57	N = 57	*p*	N = 56	N = 56	*p*	N = 58	N = 58	*p*
Symptoms - VAS									
Nasal obstruction	70.4(21.9)	29.1 (26.6)	*0.01*	71.8 (16.4)	27.5 (22.5)	*0.01*	66.8 (23.6)	37.2 (24.8)	*0.01*
Nasal discharge	40.6 (31.9)	20.5 (25.4)	*0.01*	39.8 (32.1)	24.2 (28.0)	*0.01*	42.0 (33.6)	29.5 (30.0)	*0.02*
Sneezing	32.2 (28.7)	18.6 (23.4)	*0.01*	27.8 (25.3)	13.2 (19.6)	*0.01*	26.4 (2540)	20.9 (20.9)	*0.18*
Snoring	50.3 (36.2)	22.2 (27.6)	*0.01*	53.2 (32.0)	27.4 (26.8)	*0.01*	45.8 (36.3)	32.7 (30.0)	*0.01*
Oral breathing	67.2 (28.5)	26.3 (29.7)	*0.01*	58.7 (30.8)	22.7 (25.9)	*0.01*	51.9 (31.9)	31.9 (30.5)	*0.01*
Reduced general health	47.7 (33.8)	14.1 (22.0)	*0.01*	43.4 (28.9)	13.0 (20.5)	*0.01*	40.4 (31.5)	18.4 (23.2)	*0.01*
HQOL - SNOT - 20									
Total SNOT 20	1.58 (0.78)	0.97 (0.80)	*0.01*	1.70 (0.84)	0.93 (0.71)	*0.01*	1.59 (0.83)	1.15 (0.87)	*0.01*
Subset:									
Rhinologic	1.83 (0.97)	1.18 (0.82)	*0.01*	1.82 (1.05)	1.17 (0.85)	*0.01*	1.84 (0.94)	1.44 (1.06)	*0.01*
Ear/facial	0.75 (0.75)	0.47 (0.67)	*0.01*	1.05 (0.91)	0.50 (0.58)	*0.01*	1.10 (0.86)	0.79 (0.92)	*0.01*
Sleep	2.25 (1.33)	1.33 (1.28)	*0.01*	2.23 (1.31)	1.15 (1.20)	*0.01*	2.01 (1.46)	1.43 (1.38)	*0.01*
Psychological	1.45 (1.16)	0.82 (1.10)	*0.01*	1.68 (1.07)	0.81 (0.93)	*0.01*	1.39 (1.04)	0.90 (1.03)	*0.01*
HQOL - SF- 36									
PF physical functioning	89.5 (9.89)	91.6 (12.3)	*0.02*	83.6 (15.2)	88.1 (19.3)	*0.01*	84.5 (16.9)	87.7 (18.9)	*0.14*
RP role-physical	68.3 (22.2)	75.2 (20.5)	*0.01*	61.6 (28.8)	71.2 (24.4)	*0.01*	69.3 (22.3)	72.2 (25.0)	*0.26*
BP bodily pain	71.5 (26.0)	76.9 (26.1)	*0.03*	66.0 (30.2)	71.8 (29.0)	*0.13*	68.5 (26.7)	72.7 (26.3)	*0.35*
GH general health	69.7 (21.3)	73.6 (23.8)	*0.07*	62.4 (22.5)	67.4 (23.8)	*0.02*	59.5 (23.1)	64.6 (23.5)	*0.03*
VT vitality	48.7 (19.4)	53.8 (18.5)	*0.02*	44.4 (15.6)	52.1 (16.0)	*0.01*	46.1 (18.4)	54.3 (19.8)	*0.01*
SF social functioning	80.5 (23.3)	86.2 (20.8)	*0.01*	76.8 (23.9)	80.4 (23.9)	*0.20*	80.2 (23.0)	85.6 (21.4)	*0.09*
RE role-emotional	85.7 (20.8)	90.8 (16.9)	*0.08*	81.8 (25.1)	84.3 (24.0)	*0.16*	91.4 (15.5)	91.4 (18.3)	*0.55*
MH general mental health	69.2 (12.9)	70.9 (14.4)	*0.20*	66.6 (11.3)	69.1 (12.0)	*0.04*	70.6 (10.3)	72.1 (11.0)	*0.18*
Physical health summary	74.9 (15.2)	79.3 (17.3)	*0.01*	68.5 (20.4)	74.6 (20.9)	*0.01*	70.5 (17.0)	74.3 (19.8)	*0.05*
Mental health summary	71.5 (16.7)	76.4 (17.3)	*0.01*	66.7 (17.2)	71.0 (18.4)	*0.01*	69.3 (15.1)	74.0 (17.1)	*0.01*

Abbreviations: Group 1, septoplasty; Group 2, septoplasty combined with radiofrequency therapy of inferior turbinate (RFIT); Group 3, RFIT only; VAS, Visual Analog Scale; SNOT-20, Sino-Nasal-Outcome-Test-20; SF-36, Short-Form-Health-Survey-36; pre, preoperative; post, postoperative. Data are presented in mean with standard deviation, *p*-values ≤ 0.01 are considered significant

### HQOL reported on SNOT-20 and SF-36 before and after surgery

Preoperatively the total SNOT-20 score showed no significant differences between the patient groups, but when we analyzed the subsets in SNOT-20 we found that the patients in group 3 reported worse problems in the ear/facial subset than the patients in group 1 *(p < 0.02)* (Table 2).

**Fig. 3:**
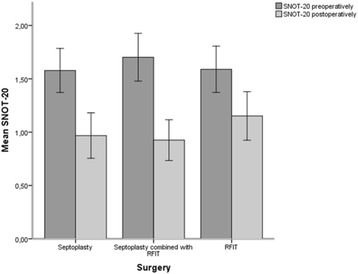
SNOT- 20 preoperatively and 6 months postoperatively. Values presented as mean, 95% CI

After surgery the total SNOT-20 score and all subset scores improved for all three patient groups (Table 2 and Fig. 3). Patients in group 1 had greater improvement in the sleep function subset than patients in group 3 *(p < 0.05*). The patients in group 2 had greater improvement in the total SNOT-20 score (*p* < 0.01) and in the sleep function- and psycologic subset compared to patients in group 3 *(p < 0.04*) (Table 3).

**Table 3:** Improvement in symptoms and HQOL 6 months postoperatively

	Improvement Group 1	Improvement Group 2	Improvement Group 3	Comparing 1 vs 2 *p*	Comparing 1 vs 3 *p*	Comparing 2 vs 3 *p*
Symptoms - VAS						
Nasal obstruction	40,5 (33.5)	44.6 (25.7)	29.5 (31.5)	0,39	*0,04*	*0,01*
Nasal discharge	19.3 (30.5)	17.4 (29.5)	12.0 (33.9)	0,86	0,15	0,20
Sneezing	12.9 (32.4)	14.4 (26.6)	5.48 (20.7)	0,40	0,14	*0,01*
Snoring	27.7 (35.0)	26.0 (28.0)	12.9 (28.3)	0,55	*0,01*	*0,01*
Oral breathing	40.1 (35.4)	37.2 (32.3)	19.9 (29.4)	0,50	*0,01*	*0,01*
Reduced general health	33.2 (33.5)	32.0 (29.1)	22.0 (32.7)	0,99	0,06	0,06
HQOL - SNOT- 20						
Total SNOT 20	0.61 (0.68)	0.78 (0.84)	0.44 (0.72)	0,30	0,15	*0,01*
Subset						
Rhinologic	0.66 (1.08)	0.65 (1.09)	0.40 (0.90)	0,95	0,28	0,11
Ear/facial	0.28 (0.62)	0.55 (0.84)	0.32 (0.88)	0,06	0,42	0,32
Sleep	0.92 (1.18)	1.08 (1.36)	0.59 (1.33)	0,82	*0,05*	*0,03*
Psychological	0.63 (0.97)	0.87 (1.01)	0.50 (1.00)	0,12	0,63	*0,04*
HQOL - SF-36						
PF physical functioning	2.17 (10.3)	4.42 (15.7)	3.17 (20.2)	*0,69*	*0,65*	*0,41*
RP role-physical	6.86 (19.9)	9.55 (24.8)	2.91 (21.2)	*0,57*	*0,48*	*0,22*
BP bodily pain	5.42 (22.8)	5.82 (27.0)	3.74 (22.5)	*0,71*	*0,38*	*0,66*
GH general health	4.49 (18.8)	5.14 (15.7)	5.10 (16.4)	*0,99*	*0,84*	*0,77*
VT vitality	5.00 (16.0)	8.00 (16.3)	8.25 (16.8)	*0,35*	*0,37*	*0,99*
SF social functioning	5.70 (18.5)	3.57 (18.9)	5.39 (24.0)	*0,66*	*0,76*	*0,50*
RE role-emotional	5.12 (20.2)	2.46 (21.8)	0.00 (19.7)	*0,64*	*0,85*	*0,43*
MH general mental health	1.96 (9.94)	2.54 (8.69)	1.43 (11.0)	*0,61*	*0,89*	*0,74*
Physical health summary	4.44 (12.9)	6.08 (16.1)	3.80 (14.0)	*0,74*	*0,59*	*0,59*
Mental health summary	4.91 (12.9)	4.33 (12.1)	4.69 (14.6)	*0,83*	*0,94*	*0,93*

Abbreviations: Group 1, septoplasty; Group 2, septoplasty combined with radiofrequency therapy of inferior turbinate (RFIT); Group 3, RFIT only; VAS, Visual Analog Scale; SNOT-20, Sino-Nasal-Outcome-Test-20; SF-36, Short-Form-Health-Survey-36; pre, preoperative; pos, postoperative. Data are presented in mean with standard deviation, p-values ≤ 0.01 are considered significant

Comparing the postoperative scores between patient groups we found no significant differences in total SNOT-20 score, but group 2 had less problems in the ear/facial subset than group 3 (Table 2).

The preoperative SF-36 summary scores, i.e. physical and mental health, between the patient groups were not significantly different (Table 2). When we analyzed the different domains of SF-36 we found that the patients in group 1 reported less problems in their general health than group 3 *(p < 0.03).* Patients in group 2 reported more problems in their emotional role and worse general mental health than patients in group 3 *(p < 0.05*).

**Fig. 4:**
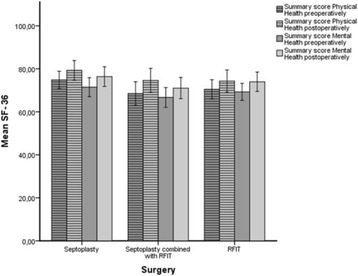
SF- 36 preoperatively and 6 months postoperatively. Values presented as mean, 95% CI

After surgery the SF-36 summary scores of physical and mental health (Table 2) improved for all three patient groups *(p < 0.05)* (Table 2 and Fig. 4). Patients in group 1 and 2 had improvement in five domains of SF-36 *(p < 0.04*), while patients in group 3 had improvement in two domains of SF-36 *(p < 0.03*). The improvement was not significantly different between the patient groups (Table 3).

Comparing the postoperative scores between patient groups we found no significant differences in the postoperative SF-36 summary scores of physical and mental health. Patients in group 1 reported better score than group 3 in the general health domain *(p < 0.03)*, while patients in group 2 reported more trouble in the role-emotional domain than patients in group 3 *(p < 0.05)* (Table 2).

### Comorbidity, previous surgery and smoking

In this study some of the patients have comorbidity such as allergy, asthma and sleep apnea and a history of previous septal or turbinate surgery. There were no significant differences in the distribution of these conditions in the groups. Subanalysis showed no difference in nasal obstruction pre- and postoperative on VAS in patients with comorbidity compared to patients without (*p* > 0.05). Patients with previous septal or ITH surgery had less improvement of nasal obstruction*(p < 0.03)* and were more bothered postoperatively with nasal obstruction *(p < 0.01)* than patients who had no previous surgery *(p < 0.01).* Regarding HQOL we found that patients with allergy preoperatively reported a worse total SNOT-20 score than patients without allergy *(p < 0.03).* Postoperatively we found no differences. Sleep apnea patients reported a worse postoperative total SNOT-20 score than patients without sleep apnea *(p < 0.01)*. Patients with asthma reported worse postoperative summary scores of physical and mental health of SF-36 than patients without asthma*(p < 0.01).* The only statistical difference regarding smoking was that smokers had worse HQOL preoperatively than non-smokers *(p < 0.03)*.

## Discussion

In this study the patients undergoing septoplasty (patient group 1), septoplasty combined with RFIT (patient group 2), and RFIT alone (patient group 3) had a fairly similar symptom burden and HQOL preoperatively. All three patient groups had a significant improvement in symptoms and HQOL after surgery (Table 2). When comparing the postoperative scores between the patient groups we find that the mean level of most of the HQOL variables were at the same level in the groups. Regarding symptoms postoperatively, the patients in group 3 reported significantly more trouble with snoring than group 1, and more trouble with nasal obstruction and sneezing than the patients in group 2 (Table 3).

Although the preoperative symptoms between patient groups were fairly similar, we note that the improvement in symptoms was significantly better for the patients in group 1 and 2 than for the patients in group 3.

We found that the patients in group 2 had an improvement in nasal obstruction of 44.6 mm on VAS, while patients in group 1 and 3 had a improvement of 40.5 and 29.5 mm respectively. Rhee et al. consider a change of 30 mm on VAS clinically meaningful [39]. Thus, based on that criterion, all three patients groups had symptom improvements that could be considered a surgical success.

According to severity of symptoms, the symptom of nasal obstruction in the two septoplasty groups changed from severe to mild symptoms after surgery (Table 2). The nasal obstruction in patient group 3 improved significantly, but were also after surgery considered to be moderate bothersome [30].

In spite of the fact that all patients report a similar symptom burden preoperatively we find that group 3 report more bothersome symptoms postoperatively than the other groups. An explanation for this could be that some of the patients in group 3 had a deviated nasal septum that was not considered clinically significant and therefore septoplasty was not done. Another explanation could be that RFIT is not as efficient in opening the nose as septoplasty or a combination of septoplasty and RFIT is. Karlsson et al. showed that concomitant inferior turbinate reduction may decrease the likelihood of revision nasal surgery [40].

The SNOT-20 was used to assess HQOL spesifically related to the sino-nasal aspects. Preoperatively we found no significant differences in the total SNOT-20 score between the patient groups (Table 2).

When we analyzed the subsets in SNOT-20 we noted that patients in group 3 reported more problems in the ear/facial of SNOT-20 than the patients in group 1. An explanation for this difference might be that more oedema of the nasal mucosa and posterior part of the inferior turbinate in the ITH patients influence on the ventilation of the ears and thus lead to more ear fullness or ear pain.

We see that our patients preoperatively report a similar total SNOT-20 score as patients with chronic rhinosinusitis, who report a total SNOT-20 score of 1.9 [31], and a worse score than healthy individuals, who report a mean SNOT-20 score of 0.4 [33]. Surgery led to an improvement in total SNOT-20 score including all subsets for all patient groups (Table 2). The improvement in total SNOT-20 score was significantly better for patients in group 2 than for patients in group 3. Regarding the subsets of SNOT-20, we found that both septoplasty groups had larger improvement in the sleep subset than group 3 (Table 3), so it is likely to believe that the larger improvement in nasal obstruction in these groups led to greater improvement in the sino-nasal aspects of HQOL.

Our findings in SNOT-20 were similar to those of other studies using SNOT-22 or other HQOL assessments of sino-nasal outcome [9, 41, 42].

Patients in group 2 had a mean improvement in the total SNOT-20 score of 0.8, while the other groups had an improvement of 0.4 and 0.6. According to Piccirillo, a change in total score of 0.8 in SNOT-20 is clinically meaningful for patients with CRS after surgery [31]. Thus, only the patients in group 2 achieved a clinically meaningful change in SNOT-20. This indicate that a combination of septoplasty and RFIT is meaningful because it seem to improve the sino-nasal aspects of HQOL more than septoplasy alone and RFIT alone. This may have implications for what kind of surgery to choose for our patients in the future.

Nevertheless, also patients in group 3 had postoperatively improved their total SNOT-20 score, and the three patient groups ended up having quite a similar total SNOT-20 score after surgery (Table 2). Therefore RFIT alone might be considered wise in patients with ITH where the nasal deviation is not clinical significant. RFIT is considered to be a safe and well tolerated procedure preserving the nasal epithelial function, with little postoperative pain, bleeding and crusting. It is a rapid procedure that can be performed under local anesthesia, allowing the patient to return to work or home immediately after treatment [43].

SF-36 was used to assess more general aspects of the patients HQOL. Preoperatively the patient groups reported a similar physical and mental health according to the summary scores of SF-36, but some of the eight domains differed slightly (Table 2).

Preoperatively the patients in group 1 reported better score in the general health domain than group 3, and patients in group 2 reported worse score in the emotional role- and general mental health domain than group 3. Thus indicating that patients with a clinically significant septal deviation combined with ITH may have worse general HQOL than patients with ITH without a clinical significant septum deviation.

After surgery all patient groups improved their physical and mental health according to the summary scores, but the improvement within domains differed between the patient groups (Table 2). Patients in group 1 had improvement in five domains; physical functioning, role-physical, bodily pain, vitality and social functioning. Patients in group 2 had improvement in five domains; physical functioning, role- physical, general health, vitality and general mental health. Patients in group 3 had impovement in the general health and vitality domain. This may indicate that the patients in group 1 and 2 had greater improvement in general HQOL than patients in group 3.

The improvement in some domains may partly be influenced by the worse preoperatively outset. Further more there could be a ceiling effect in the questionnaire indicating that patients in group 3, who had extreme high scores preoperatively in the emotional role domain, could not respond with even more extreme high scores.

In spite of these influences, our results also imply that septoplasty and septoplasty combined with RFIT improved the general HQOL more than only RFIT. Our findings in improvement in general aspects of HQOL after septoplasty is supported by others [42], but not by all [9, 41]*.*

We found that all patient groups reported similar general HQOL postoperatively, except for patients in group 1 who reported better HQOL in the general health domain than group 3 like they did preoperatively, and that the patients in group 2 reported worse HQOL in the role- emotional domain postoperatively than group 3 as they did preoperatively. This might indicate that all three surgical procedures influence these two aspects of SF-36 equally.

None of our groups of patients reached the same level in general aspects of HQOL as healthy people [44]., Our sub-analysis showed that patients with allergy report worse HQOL on SNOT-20 before surgery than non-allergic patients. After surgery we found no differences. Nevertheless, treatment of allergy is important also after surgery. The patients with sleep apnea reported postoperatively worse HQOL in SNOT-20 score than patients without sleep apnea *(p < 0.01).* The same results were found for the asthma patients regarding postoperative summary scores of physical and mental health in SF-36 *(p < 0.01),* thus more or other treatment [45] than nasal surgery should be considered for these patients. Patients with previous surgery were more bothered with nasal obstruction after surgery (p < 0.01) which may indicate that surgery in these patients is more challenging.

The major strength of this study is the prospective design and the high follow-up rate (81%). This study has some limitations. We did not randomize the patients to treatment groups. We wanted this study to reflect the daily practice in an out patient clinic. We used the SNOT-20 to evaluate sino-nasal quality of life because we did not have a validated translation of the SNOT-22 questionnaire at the onset of the study. SNOT-20 lack questions about nasal obstruction and sense of smell in the first subset, the three other subsets are equal with SNOT-22. We have compensated for this by evaluating the nasal obstruction on VAS which we know have a strong correlation to nasal resistance [46]. However, the lack of postoperative difference in SNOT-20 between patient groups may be caused by the lack of question about nasal obstruction. Using SNOT-22 may have led to a slightly different outcome regarding HQOL.

We used two different devices for RFIT and one could argue that this might influence our results. The Celon ProBreath® was used in the patients in group 2 and the Sutter system BM-780 II was used on all patients in the group 3. A review comparing different surgical techniques for bilateral ITH reduction reported no significant difference in nasal obstruction using either microdebrider-assisted turbinoplasty or multiple types of radiofrequency devices [16].

## Conclusion

We have shown that surgical treatment of nasal obstruction leads to less symptoms and better HQOL for all three patient groups. Patients treated with septoplasty alone or septoplasty combined with RFIT achieved a better improvement in symptoms, and patients treated with septoplasty combined with RFIT also achieved a better improvement in HQOL than patients treated with only RFIT. Nevertheless, comparing the postoperative scores we find that all patient groups reach about the same level of HQOL. Regarding symptoms, the patients in group 2 reported less nasal obstruction postoperatively than patients in group 3 which may indicate that a combined procedure of septoplasty and RFIT is better than RFIT alone to treat nasal obstruction. Furthermore, revision cases, patients with sleep apnea and asthma patients seem to have poorer outcome after surgery than other patients. Both disease specific and general QOL instruments add valuable information for identifying factors influencing outcome.

## Additional file

Additional file 1: Spreadsheet that include information from the patients about symptoms given on VASs and HQOL given on SNOT-20 and SF-36. (XLS 306 kb)
Additional file 2: Response letter. (DOCX 18 kb)

## Abbreviations

HQOL: Health related quality of life
ITH: Inferior turbinate hypertrophy
RFIT: Radiofrequency therapy of inferior turbinate
SD: Standard deviation
SF-36: Short-form-health-survey-36
SNOT-20: Sino-nasal-outcome-test-20
VAS: Visual analogue scale
