# Supplementary material for: A comparison of symptoms and quality of life before and after nasal septoplasty and radiofrequency therapy of the inferior turbinate
Source: BMC Ear Nose Throat Disord. 2018 Jan 26;18:2. doi: 10.1186/s12901-017-0050-z (PMC5787231; doi:10.1186/s12901-017-0050-z)
Supplement: Additional file 2: — Response letter. (DOCX 18 kb) [file 12901_2017_50_MOESM2_ESM.docx]

Dear Editor and Reviewers	Response letter ENTD-D-17-00003R2	12.06.2017

Thank you for the opportunity of having our manuscript reviewed for publication in BMC Ear, Nose and Throat Disorders.

Response to Editors comments:

*1) We note that you are reporting a clinical trial but have not cited a trial registration number:*

This study is not a clinical trial, it is an observational study based on registry data.

We have consulted the Unit for Applied Clinical Research, NTNU and according to the [ICMJE FAQs](http://www.icmje.org/about-icmje/faqs/clinical-trials-registration/) definition of a clinical trial: “Purely observational studies (those in which the assignment of the medical intervention is not at the discretion of the investigator) will not require registration.”

<http://www.icmje.org/about-icmje/faqs/clinical-trials-registration/>

2) *On the title page, please include the email addresses of all authors.*

Email addresses are now included of all authors

3) *Please use a Declarations Heading in the Declarations section, which is found after the list of Abbreviations*

Declaration heading is now in page 16, line 426

4) *Please remove the titles/captions/numbers included in the figures and place them at the end of the main manuscript, after the References, in a section ‘Figures’ where you list the following information: * Title of data (e.g. Figure 1) and * Description of data.*

Figures are now without titles and a section “Figures” with list of “Title of data and Description of data” are placed after the references at page 21

We hope these revisions makes it possible to get the article published in BMC Ear, Nose and Throat disorders.

Yours sincerely Ann Helen Nilsen
